# The therapeutic effects of physical treatment for patients with hereditary spastic paraplegia: a narrative review

**DOI:** 10.3389/fneur.2023.1292527

**Published:** 2023-11-29

**Authors:** Armando Di Ludovico, Francesca Ciarelli, Saverio La Bella, Giovanna Scorrano, Francesco Chiarelli, Giovanni Farello

**Affiliations:** ^1^Department of Pediatrics, University of Chieti “G. D’ Annunzio”, Chieti, Italy; ^2^Department of Pediatrics, University of L’Aquila, L’Aquila, Italy

**Keywords:** neurology, rehabilitation, physiotherapy, hereditary spastic paraplegia, physical therapy

## Abstract

**Background:**

Hereditary spastic paraplegia (HSP) encompass a variety of neurodegenerative disorders that are characterized by progressive deterioration of walking ability and a high risk for long-term disability. The management of problems associated with HSP, such as stiffness, deformity, muscle contractures, and cramping, requires strict adherence to recommended physiotherapy activity regimes. The aim of this paper is to conduct a critical narrative review of the available evidence focusing exclusively to the therapeutic advantages associated with various forms of physical therapy (PT) in the context of HSP, emphasizing the specific benefit of every distinct approach in relation to muscle relaxation, muscle strength, spasticity reduction, improvement of weakness, enhancement of balance, posture, walking ability, and overall quality of life.

**Methods:**

To conduct a literature review, the databases PubMed, Scopus, and DOAJ (last access in June 2023) were searched.

**Results:**

The PubMed search returned a total of 230 articles, Scopus returned 218, and DOAJ returned no results. After screening, the final list included 7 papers on PT treatment for HSP patients.

**Conclusion:**

Electrostimulation, magnetotherapy, hydrotherapy, PT, robot-assisted gait training, and balance rehabilitation have the potential to increase lower extremity strength and decrease spasticity in HSP patients.

## 1 Introduction

**Figure 1:**
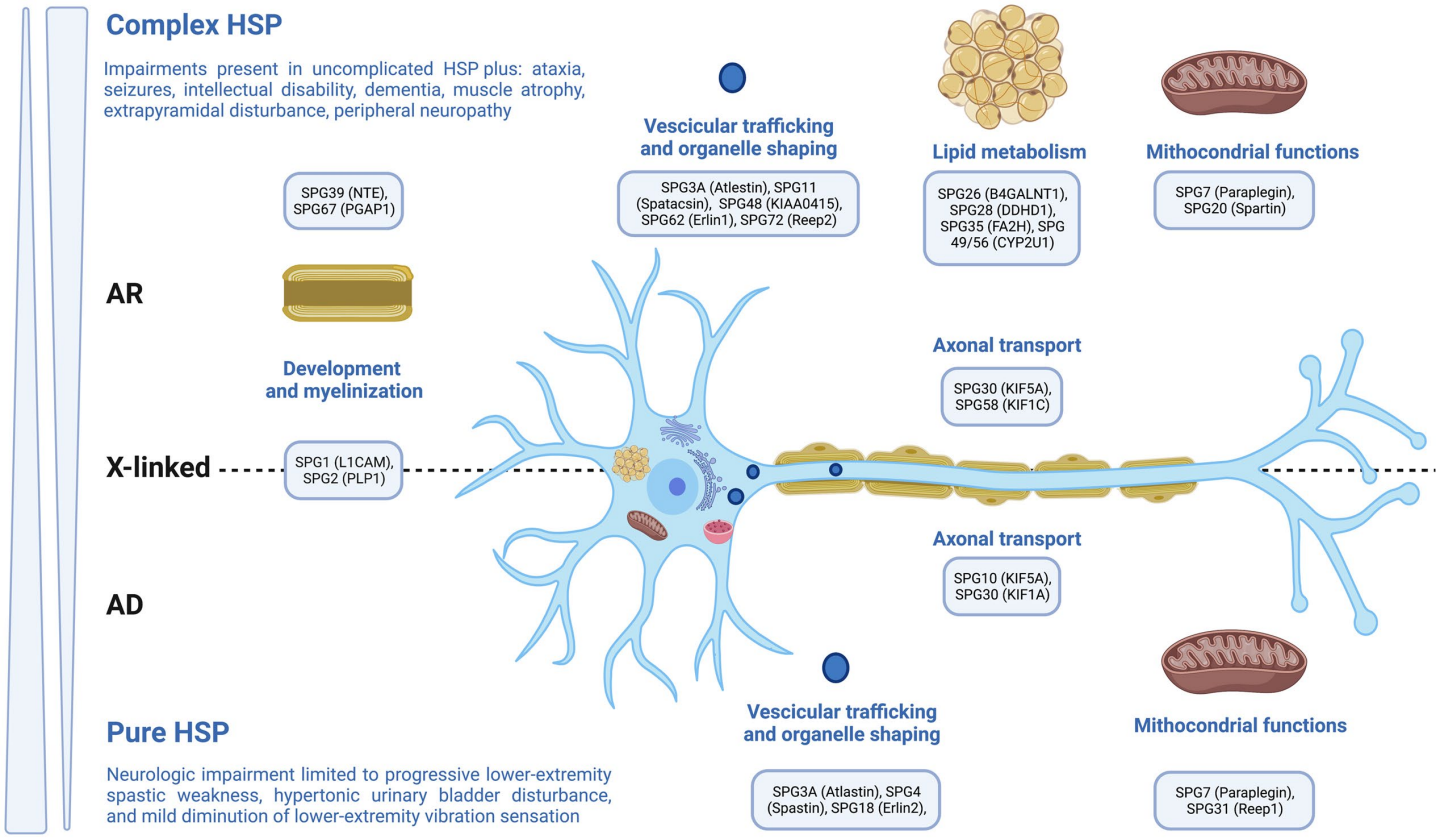
Genetics in Hereditary Spastic Paraplegia.

Genetic neurodevelopmental disorders (NDDs) encompass a range of monogenic illnesses that exhibit increasing clinical and genetic diversity. These disorders are defined by the inconsistent and atypical development of language, motor skills, cognition, and behavior. Additionally, individuals with genetic NDDs often experience neurological comorbidities, such as epilepsy and movement abnormalities (1–8). Hereditary Spastic Paraplegia (HSP) is a collection of hereditary disorders that impact the neurological system, resulting in the manifestation of spasticity and muscular weakness specifically in the lower extremities (9). The previously mentioned disorders are distinguished by the degeneration of the longest axons present in the corticospinal tract (Figure 1). Currently, physiotherapy is among the recommended treatments, and it would be highly beneficial to prioritize the implementation of our knowledge regarding the most effective approach to pursue (9). The current literature has provided a comprehensive review of HSP, encompassing its epidemiology, pathophysiology, clinical symptoms, and several treatment approaches, which include both pharmaceutical and non-pharmacological therapies. Physiotherapy has been discussed as a therapeutic option among the non-pharmacological interventions. The aim of this paper is to perform a comprehensive narrative review of existing evidence, specifically examining the therapeutic benefits of different physical therapy modalities in the context of HSP. This review will emphasize the distinct advantages of each approach in terms of muscle relaxation, muscle strength, reduction of spasticity, improvement of weakness, enhancement of balance, posture, walking ability, and overall improvement in quality of life.

## 2 Methods

A comprehensive literature review was conducted using the databases Medline/Pubmed, Scopus, and DOAJ, with the most recent access occurring in June 2023. The search focused on the topic of physiotherapy in individuals with HSP, covering the period from 2002, to June 1, 2023. The key terms employed in the literature search encompassed the following: ‘physiotherapy’, ‘physical therapy’, ‘hereditary spastic paraplegia’, ‘rehabilitation’, and ‘HSP’. We incorporated primary research studies and literature reviews that were pertinent to our research aims. Exclusions were made for articles, duplication, and reports published in languages other than English. The PubMed database yielded a cumulative count of 230 scholarly papers, while Scopus database provided a total of 218 articles. However, the DOAJ database did not yield any findings. The titles and abstracts were examined and redundant entries were removed. Subsequently, the remaining 32 papers underwent a further screening process, wherein 25 of them were excluded due to their failure to meet the inclusion criteria, notably pertaining to the focus on physiotherapy for patients with HSP. The ultimate compilation consisted of seven publications pertaining to physiotherapeutic interventions for patients diagnosed with HSP, as indicated in Table 1. The studies that were selected investigated the application of electric stimulation, magnetotherapy, hydrotherapy, physical therapy, robot-assisted gait training, and balance rehabilitation in individuals diagnosed with HSP. Ultimately, the data was consolidated in a coherent order, using the valuable insights of the senior authors.

**Table 1:** Characteristics of included studies.

Study	Participants	Interventions	Outcomes
Ardolino (9)	11 patients with HSP (mean age 37.3 ± 8.1 years; 6 men and 5 women; 8 affected by spastin/SPG4,1 by atlastin1/SPG3a, 1 by paraplegin/SPG7 and 1 by ZFYVE26/SPG15)	Subjects were studied before and after anodal (group A) and sham (group B) tsDCS	Group A: improvements Ashworth score, 5MTW, and SPRS
Zeigelboim (10)	40 patients with HSP (unspecified age; unspecified gene variants)	To determine the benefits of vestibular rehabilitation involving virtual reality by comparing pre-and post intervention assessments	Delay in the progression of the disease, providing a personalized, accessible, low-cost treatment option. Promotion of enthusiasm and motivation of the patient during continuous sessions
Antczak (11)	15 patients with HSP (mean age 43.7 ± 10.6 years; 5 women and 10 men; AMN: 6 patients with ABCD1 gene variants; HSP: 1 patient with HSP3A, 1 with HSP7, 5 with unspecified HSP pure, and 2 with unspecified HSP complex variants)	Five sessions of bilateral 10 Hz rTMS over primary motor areas of the muscles of lower extremities and five sessions of similar sham stimulation	Improvements in weakness and spasticity of lower extremities (Ashworth scale)
Sato (12)	1 patient with HSP (41 year old-man; SPG 11 gene with autosomal recessive mutation)	20 min/session of 2 days/week for 12 weeks vs. 6 days/week for 12 weeks of squatting, kneeling position exercises, and a motion exercise of taking a bath	Prevention of further regression of the lower limb function of the patient with a complicated form of HSP, and prevention of decreasing in the ability of the patient to perform ADL (Barthel index). Improved muscle strength (grip force and quadriceps)
Bertolucci (13)	13 patients with HSP (mean age 46.3 ± 8.9; 7 women and 6 men; 6 patients with SPG7, 5 with SPG4, 1 with SPG5, and 1 with SPG11 gene variants)	Pre- and post-6-week robotic-aided gait training protocol	Post-training: Improvements in balance (Berg scale) and walking ability (10mWT, 6mWT) and in quality of life (SF-36)
Zhang (14)	9 patients with HSP (unspecified age, sex, and gene variants)	Pre- and post-hydrotherapy gait analyses	Increased walking speed, step length, and increased ability to perform compensatory strategies in post-hydrotherapy clinical gait analysis

10mWT, 10 meter walking test; 5mWT, 5 minutes walking test; 6mWT, 6 minutes walking test; ADL, activities of daily living; HSP, hereditary spastic paraplegia; rTMS, repetitive transcranial magnetic stimulation; SF-36, Short Form 36; SPRS, spastic paraplegia rating scale; tsDCS, transcutaneous spinal direct current stimulation.

## 3 Results of physiotherapy approaches in HSP

### 3.1 Electric stimulation

The literature search yielded one crossover research involving sham-controlled electric stimulation in HSP. Ardolino et al. conducted a study in which they recruited a sample of 11 patients diagnosed with HSP (eight affected by spastin/SPG4,1 by atlastin1/SPG3a, 1 by paraplegin/SPG7 and 1 by ZFYVE26/SPG15) with the purpose of investigating the effects of spinal direct current stimulation (tsDCS). The study followed a sham-controlled crossover design and included six male and five female participants, with a mean age of 37.3 ± 8.1 years. A total of eight individuals, consisting of four males, were affected by Spastin/SPG4. Additionally, one male individual was impacted by atlastin1/SPG3a, one female individual was affected by paraplegin/SPG7, and one male individual was affected by ZFYVE26/SPG15.4. The participants were administered spinal direct current stimulation at a current intensity of 2.0 mA for a duration of 20 min. This stimulation was delivered by a stimulator that was connected to a pair of rectangular electrodes. One electrode was positioned above the spinous process, specifically between the 10th and 12th vertebrae, while the second electrode was placed over the right shoulder (9). In order to elicit a significant level of blindness throughout the intervals, the electrical current was gradually increased for a duration of 30 s at the initiation of the tsDCS procedure, and afterwards reduced for a duration of 30 s at its conclusion. The tsDCS method was concealed from the patients, who were unable to differentiate between the anodal and placebo circumstances. The individuals were investigated prior to and following anodal and sham tsDCS. In a cross-over design, each patient received either anodal (A) or placebo (S) therapy. In order to prevent carry-over effects, each patient participated in two separate experimental conditions at least 3 months apart. Five days per week, twice per day for 20 min, each anodal or sham session occurred. There were no observed pharmacological alterations during the week of tsDCS or the 3 months interval between trial sessions. In addition, no further treatment interventions (such as physical therapy) were administered during the study period. The operation was without problems and was well tolerated by all patients. Regarding the scores on the Ashworth scale for the lower limb, it was seen that the group receiving anodal stimulation exhibited superior performance compared to the group receiving sham stimulation. This difference was notably evident up to 2 months after the stimulation ended, as indicated by the statistical significance at T1 (*p* = 0.0137) and T4 (*p* = 0.0244). The results of the study indicate that patients exhibited significant enhancements in hip flexion (T4: *p* = 0.016) and knee extension (T2: *p* = 0.0156; T3: *p* = 0.0078; T4: *p* = 0.0039) across many subcategories, including hip extension, leg abduction, knee flexion, knee extension, ankle flexion, and ankle extension. Nine patients underwent the clinical evaluation, which included the Five-Minute Walking Test and the Spastic Paraplegia Rating Scale (SPRS). The results did not show a statistically significant difference in scores. However, there was a noticeable tendency towards improvement in the group receiving anodal stimulation, as indicated by the Five Minutes Walking test (*p* = 0.072, Friedman analysis). Conversely, there was no observed improvement in non-motor symptoms, as assessed by the Spastic Paraplegia Rating Scale (*p* = 0.80) (9). There is a growing number of research indicating that transcranial direct current stimulation (tsDCS) has the potential to reduce cortical, corticospinal, and spinal motor output in human subjects. However, the precise processes underlying this phenomenon have yet to be fully elucidated (15). The inhibitory effects of anodal transcranial direct current stimulation (tsDCS) on the gamma system in humans can be attributed to the modulation of both alpha and gamma motor neuron activity by spinal stimulation, as observed in animal studies (16). Moreover, the modulation of interneuronal excitability with transcranial direct current stimulation (tsDCS) can potentially lead to a decrease in spasticity by inducing pre-synaptic inhibition and post-activation depression (17). In this study, the effects of tsDCS on HSP patients were initially investigated. The results validated previous studies on chronic stroke and spinal cord injuries, demonstrating the efficacy of tsDCS in treating spasticity. The findings may therefore contribute in the development of more focused clinical applications. The integration of transcranial and spinal stimulation techniques has significant promise in the expansive domain of movement disorders, offering an opportunity of enhancing motor recovery, as evidenced by observations in animal studies (18). Future trials should incorporate more frequent stimulation cycles due to the fact that the advantage can last up to 2 months (T4) before clinical scores begin to fall (9).

### 3.2 Magnetotherapy

The current literature lacks definitive guidelines about the specific modalities and optimal time of Magnetic Stimulation for HSP. Antczak et al. recruited a sample of 15 individuals diagnosed with Adrenomyeloneuropathy (AMN) (6 patients with ABCD1 gene variants) as well as simple and complex kinds of HSP (1 patient with HSP3A, 1 with HSP7, 5 with unspecified HSP pure, and 2 with unspecified HSP complex variants). The participants had a mean age of 44.8 ± 10.1 years, consisting of five females and ten males. Each individual exhibited chronic, progressively worsening spastic paraparesis, which was clinically characterized by impaired walking and had a notable effect on their daily functioning, as reported by the patients. Bilateral application of transcranial magnetic stimulation at a frequency of 10 Hz was administered to the primary motor areas (PMA) associated with the muscles of the lower limbs. The determination of the specific sites for stimulation and the computation of the motor threshold (MT) were crucial for the left and right abductor hallucis (AH) muscles. The AH and proximal phalanx of the hallux were encompassed using recording electrodes (11). In the case of rTMS, the stimulation intensity was established at 90% of the resting motor threshold (RMT). However, in instances when patients had significant spasticity and were unable to maintain complete muscular relaxation, the stimulation intensity was set at 90% of the active motor threshold (AMT). The determination of the MT value was conducted utilizing the relative frequency method, as prescribed in the IFCN recommendations. This approach involves identifying the lowest magnetic field strength necessary to elicit motor responses of a specific amplitude in a minimum of five out of ten stimuli (19). During each session, a total of 1,500 magnetic pulses were administered to the lower limb muscles, resulting in a cumulative total of 3,000 pulses each session (19). Using a 10 Hz frequency and 7.5 s trains, 40 stimulations were administered to each hemisphere. Each train had seventy-five stimuli. At 56 s intervals, trains were spaced apart. During the process of stimulation, the participants assumed a semi-recumbent position (11). During the simulated stimulation, the coil was positioned in a perpendicular orientation to the scalp by rotating it 90 degrees towards the posterior direction. Apart from this adjustment, the technique remained unchanged. The aforementioned strategy effectively decreases the magnetic field by a range of 67–73%, resulting in the elimination of any potential biological impacts. However, it is worth noting that this reduction in magnetic field does not have a major impact on the auditory perception of the clicking sound or the sensory experiences, which remain comparable to those observed during active stimulation (20). Every participant underwent both real and sham stimulations in a randomized sequence. During the course of the trial, patients adhered to their regular spasticity medication and physiotherapy regimen. The majority of patients administered baclofen adhered to a thrice-daily dosing regimen, with the second dose administered just before their repetitive transcranial magnetic stimulation (rTMS) session, typically around 1:00 p.m. A single patient, specifically a 29 years-old male, discontinued treatment as a result of experiencing a seizure. A total of 14 patients who maintained their enrollment successfully finished the research. Abnormalities were observed in every subject. Patients diagnosed with adrenomyeloneuropathy (AMN) and a majority of other patients demonstrated a reduction in amplitude of motor evoked potentials (MEP), afterwards accompanied by an extension of central motor conduction time (CMCT). Following the implementation of effective stimulation, there was observed enhancement in the muscular strength of both the proximal and distal muscles in the lower extremities. Additionally, a decrease in the stiffness of the proximal muscles was noted. During the subsequent assessment, there were still discernible alterations in spasticity. The application of repetitive transcranial magnetic stimulation (rTMS) did not yield any discernible benefits in the 10-Meter Walk Test (10MWT). Following a deceptive stimulus, there was an absence of any discernible alteration (11). A *post hoc* evaluation was conducted to examine the correlation between motor threshold (MT) and the therapeutic effects, taking into account the diverse range of MT values and rTMS intensities observed in the study group. This analysis was motivated by prior research that has established a connection between changes in MT and the pathology of HSP (9). The study revealed noteworthy associations between the alterations in strength and spasticity resulting from active repetitive transcranial magnetic stimulation (rTMS) and the average motor threshold (MT) of the 14 patients who effectively finished the study. These alterations encompassed changes in both proximal and distal muscle strength following rTMS, as well as changes in proximal muscle spasticity after rTMS and during the follow-up period. In instances when no motor evoked potentials (MEPs) were elicited, the patient’s motor threshold (MT) value was adjusted to 100% of the maximal stimulator output. The therapy of proximal muscles in the lower limbs may yield more favorable outcomes due to the superficial locations of the corresponding cortices. Given the current findings, further investigation is warranted to closely monitor cortical excitability and neurophysiological indicators of spasticity, alongside implementing therapy with extra sessions and a larger cohort of patients. Additionally, the impact of rTMS in conjunction with other treatments designed to improve gait performance should be investigated. The research on the rehabilitation of gait with rTMS employed a variety of rTMS protocols, session counts, and coil types, necessitating comparison investigations to optimize this method of treatment (21).

### 3.3 Hydrotherapy

A preliminary investigation was conducted to assess the effectiveness of hydrotherapy as a therapeutic intervention for enhancing locomotor function in individuals diagnosed with late-onset HSP. The study done by Zhang et al. aimed to assess the impact of hydrotherapy on gait characteristics in a sample of eleven patients diagnosed with late-onset HSP. One person withdrew due to pneumonia, and another could not be reached for the final evaluation. Before the first hydrotherapy session, one participant began to take baclofen orally. Throughout the duration of the trial, other participants’ medical care remained unchanged. Each participant participated in a 10 weeks hydrotherapy program after receiving a biomechanics evaluation. The treatment started with an initial group hydrotherapy session, consisting of two small groups. This was then followed by a period of five weeks, during which participants received individual hydrotherapy twice a week. Subsequently, another group session was conducted, followed by an additional 5 weeks period of individual hydrotherapy. Each session had a duration of 45 min. After the hydrotherapy session, a final biomechanics examination was carried out (14).

None of the participants exhibited spasticity beyond a score of 1 on the Modified Ashworth Scale (MAS), indicating that the affected body part could only be moved in flexion or extension with a catch and release mechanism or with minor resistance at the end of the range of motion (22). Two subjects demonstrated a Modified Ashworth Scale (MAS) score of 1, indicating the presence of spasticity in their adductor muscles, knee flexor muscles, knee extensor muscles, and plantarflexor muscles. Out of the remaining seven subjects, six individuals (all with a Modified Ashworth Scale score of 1) demonstrated bilateral stiffness in the plantarflexor muscles of the right ankle. The spatio-temporal measures of the gait cycle before and after hydrotherapy revealed a statistically significant 11% improvement in walking speed (0.85 m/s before to 0.94 m/s after, *p* < 0.05) (14). The walking pace of a single subject exhibited a decline, ranging from 0.03 to 0.025 m/s, whilst the remaining six participants demonstrated improvements. Furthermore, it was observed that there was a tendency for longer strides, with a value of *p* of 0.07. Based on the presented graphs, it can be observed that the gait trajectories of individuals with HSP were generally similar to those of the control group across all joints. The group diagnosed with HSP exhibited higher variability in the flexion/extension plane, as indicated by a standard deviation (STD) of 7.36 for HSP pre-therapy, compared to 6.60 for the control group. Additionally, the HSP group demonstrated increased hip internal rotation at initial contact, reduced knee flexion and extension during both stance and swing phases, and decreased ankle dorsiflexion during the swing phase (14). Following the application of hydrotherapy, there was a significant decrease in the range of rotation at the hip (*p* < 0.01), knee (*p* < 0.01), and ankle (*p* = 0.07) as compared to the mechanics observed prior to the therapy. When comparing the group diagnosed with HSP to the control group, a noticeable tendency towards reduced valgus to varus motion (*p* < 0.01) and significantly lower dorsiflexion (*p* < 0.01) was seen. Following the therapeutic intervention, there was a notable decrease in the rotational range of motion in comparison to the pre-treatment condition (*p* < 0.01). However, no significant changes were observed in the sagittal and frontal planes. While the control group exhibited little changes in kinematic parameters, it was observed that there was a significant decrease in ankle dorsiflexion (*p* < 0.05) in the experimental group (14). Prior to the swing, ankle plantarflexion was limited. There was reduced force at the ankle as a result of the decreased angular acceleration and the maintenance of normal moments. Although rotational range of motion tended to decrease after hydrotherapy (*p* = 0.07), similar to the findings at the hip and knee, there was minimal improvement. According to the average normalized joint kinetics profile, the participants with HSP showed comparable joint moment curves in relation to the timing of the gait cycle as compared to the controls. During standing, participants with HSP demonstrated decreased hip abduction moment, increased hip extension moment, and decreased hip flexion moment compared to controls (14). Following the therapeutic intervention, there was a significant increase in the aberrant extension moment at initial contact, with the average value rising from 0.42 N m to 0.88 N m (*p* < 0.01). This deviation from the control group was further amplified. No significant disparities in knee moments were seen between individuals with hamstring strain injury (HSP) and the control group. Following the therapy session, individuals diagnosed with high sensitivity to pain (HSP) had a notable reduction in external rotation moments, extension moments during push-off, and extension moments upon initial knee contact (decreasing from 0.8 to 0.42 N m, *p* < 0.1). This observation represents an extra departure from the established control values. There were no significant differences seen in the moments of the ankle between the control group and the pre- and post-therapy periods (14). The lack of a twofold augmentation in the ankle moment, as would have been anticipated in the presence of greater spasticity, further supported the conclusion made by the Modified Ashworth Scale (MAS) that ankle spasticity was of a moderate nature (23). The findings suggested that individuals diagnosed with HSP employ compensatory strategies in their gait patterns in order to optimize walking efficiency. Nevertheless, it seems that the benefits were mostly influenced by a rise in the compensatory approach of internal rotation/stiff knee, rather than an enhancement in the dynamics of walking. This is evident as individuals with HSP reported an elevated walking pace subsequent to undergoing hydrotherapy treatment. The observed increase in walking speed could perhaps be attributed to improvements in physical strength, flexibility, or self-confidence. This implies that the implementation of a therapeutic intervention aimed at enhancing both range of motion and muscular strength has the potential to positively impact the walking speed of individuals within this specific demographic (14).

### 3.4 Physical training

Only a few case reports and uncontrolled studies were found to document the effectiveness of physical therapy for HSP patients. Samuel et al. observed functional improvement in HSP patients following their original six-workout-per-week exercise schedule (24). In contrast to the approach employed by Samuel et al., Sato et al. (12) implemented a rehabilitation regimen for a 41 years-old male with HSP (SPG 11gene with autosomal recessive mutation) that consisted of two sessions per week. This frequency was seen to be less than half of the frequency utilized in Samuel’s study. A program characterized by infrequent exercise demonstrated the ability to maintain posture, activities of daily living (ADL) capabilities, and muscular strength. The results of this study indicate that a low frequency of exercise is likely to be adequate for those with HSP to maintain their physical function. At the commencement of the intervention, the grip strength and quadriceps strength were recorded as 28.0/29.3 kgf. After a span of 3 months following the intervention, it was seen that the grip force measured 32.0/31.7 kgf, while the quadriceps strength measured 26.3/28.0 kgf. These findings suggest that the muscle strength remained consistent throughout the duration of the intervention. Furthermore, the scores for each individual item on the Barthel Index remained unchanged. Once every week, he was still able to bathe, and there were no accidents in the bathtub at home. The standing posture and tempo did not noticeably change (12). Regarding LASER, TECAR, ultrasound, and manual therapy in HSP patients, the scholarly literature remains silent.

### 3.5 Robot assisted gait training

There are already a variety of robotic locomotion devices available to assist individuals with various neurological conditions in training their gaits. One uncontrolled study examined the efficacy of an intensive training program with robotic help for the treatment of individuals with pure HSP. Bertolucci and colleagues conducted a study in which they enrolled a total of 13 individuals diagnosed with uncomplicated HSP (6 patients with SPG7, 5 with SPG4, 1 with SPG5, and 1 with SPG11 gene variants). The sample consisted of 7 female and 6 male participants, with ages ranging from 31 to 62 years. The mean age of the participants was calculated to be 46.3 ± 8.9 years. All participants underwent a 6 weeks rehabilitation program with the Lokomat device, consisting of three sessions each week. The therapy session was began by a qualified physician who applied maximum assistance (100 percent force steering) to both legs, allowing the patient to walk at their highest allowable pace. Additionally, a body weight support (BWS) of 40 percent was provided (13). Subsequently, the therapist proceeded to systematically reduce the level of assistance applied to both legs, ensuring that it remained above 75%. This adjustment was made in accordance with the patient’s muscular tone and gait quality, specifically focusing on factors such as satisfactory knee control during the stance phase, symmetrical stepping patterns, absence of knee buckling, and absence of toe drag. During the treatment regimen, adjustments were made to the duration, distance, and pace of the exercises in order to enhance the workload, optimize patient exertion, and assess the adaptation to the training (13). The time points selected for outcome measurement were the week preceding the initiation of the treatment (T0), the conclusion of the treatment (T1), and a 2 months follow-up period. Following the completion of the treatment (T1), there was a statistically significant improvement observed in the Berg Balance Scale (mean score of 46.8 ± 10.7 at T0 compared to 50.5 ± 9.6 at T1), the 10 meters Walking Test (mean time of 13.3 ± 9.9 s at T0 compared to 11.8 ± 8.9 s at T1), and the 6 Minutes Walking Test (mean distance of 323.8 ± 118.0 meters at T0 compared to 365.7 ± 122.1 meters at T1). However, no significant changes were observed in the Physiological measures. Based on the Modified Ashworth Scale, there was no observed alteration in muscle tone. There was a significant improvement observed in the physical role (55.8 ± 39.7 at T0 compared to 78.8 ± 37.9 at T1), social function (66.2 ± 24.5 at T0 compared to 79.5 ± 18.2 at T1), emotional role (66.3 ± 30.5 at T0 compared to 79.5 ± 18.2 at T1), and mental health (67.4 ± 20.4 at T0 compared to 74.5 ± 23.3 at T1) (13). Based on the findings of the Hospital Anxiety and Depression Scale, it was observed that anxiety exhibited a considerable decrease from T0 (mean score of 5.6, SD of 4.8) to T1 (mean score of 3.7, SD of 4.9). Similarly, depression demonstrated a slight drop from T0 (mean score of 6.2, SD of 4.9) to T1 (mean score of 4.8, SD of 5.4). There were no significant differences observed between the functional tests and psychological assessments during the 2 months follow-up as compared to the initial assessment (T1). Robotic gait training has been found to be a durable intervention for individuals with uncomplicated HSP, resulting in improved balance and mobility. Moreover, this intervention has demonstrated a favorable impact on the overall quality of life of affected individuals (13).

### 3.6 Balance rehabilitation

Both intrinsic and extrinsic factors have the potential to disrupt the equilibrium of a system, such as impaired anticipatory postural adjustment to self-initiated movement, which can be caused by circumstances such as an icy surface. In patients diagnosed with HSP, there is evidence to suggest that intrinsic factors play a substantial role. One example of a potential consequence is the occurrence of retropulsion during a sit-to-stance transfer or the manifestation of a quick knee extension during the single-stance phase of stride, which might pose a risk to postural stability (25). During locomotion, compensatory trunk motions to increase step length and foot clearance may also produce imbalance issues. After a natural disruption of equilibrium, it is necessary to restore equilibrium by means of a reaction. There are three basic tactics that humans utilize in order to restore their equilibrium throughout the process of walking. (1) The hip approach entails the rotational movement of the upper body segments around the Center of Mass (CoM) (26); (2) the procedures for adjusting foot location and positioning, and (3) the ankle tactics that are prioritized above hip ways in situations where all three options are available for balance recovery. These scenarios include walking on a narrow beam, executing tasks under time constraints, or being impacted by a neurological condition (25). Individuals diagnosed with HSP may encounter challenges in utilizing the ankle strategy for balance correction (25). This can be attributed to various factors, including spasticity in the lower extremities, particularly in the calf muscles, weakness in the ankle dorsiflexors, deformities in the ankle-foot region such as pes equinus or pes equinovarus, as well as sensory or cerebellar ataxia (25). In response to this situation, those undergoing treatment exhibit a growing dependence on the foot placement approach. In response to this situation, those undergoing treatment exhibit a growing dependence on the foot placement approach (27). In instances where mediolateral changes are required, it becomes imperative to augment the step width. In individuals with more pronounced symptoms of HSP, the use of hip strategies may be essential for preserving equilibrium when walking, thereby accounting for the observed rise in trunk movements (25). Patients with this condition may exhibit ataxia, proximal lower-extremity spasticity, such as in the hip adductors, and/or observable postural sluggishness. The absence of measurement of truncal ataxia during the instrumented gait evaluation precludes its complete exclusion as a potential alternative reason for heightened trunk movements, although its low probability (28). A research study was conducted on a group of nine individuals who were in good health (28). The participants were subjected to the use of immobilizing casts on both feet and ankles. This intervention aimed to artificially restrict the recruitment of ankle strategy in order to examine its impact on trunk mobility. The findings of the study indicated that the use of bilateral foot- and ankle-immobilizing casts resulted in an observable increase in trunk mobility. When comparing the act of walking while wearing a cast to walking while wearing thin shoes, it was observed that trunk latero-flexion exhibited an increase during the stride in the cast condition (28). In a similar vein, it has been observed that the utilization of inflexible ankle-foot orthoses in children diagnosed with cerebral palsy restricts the application of ankle strategies, resulting in an augmentation of lateral trunk movements in comparison to ambulation without any footwear (29) and to walking in shoes without orthotics (30). The prevalence of toe walkers in our patient population, who exhibit significantly heightened trunk movements, can be attributed mostly to the absence of ankle strategies, which necessitate plantigrade foot contact for optimal execution (25). The evident increase in mechanical energy consumption linked to increased joint motions, leading to elevated metabolic expenses, represents a drawback of heightened trunk mobility during the act of walking. Individuals of a younger age who have HSP may exhibit a greater degree of trunk movement during walking, despite the accompanying higher energy expenditure. Conversely, elderly individuals with HSP tend to find this level of exertion excessively fatiguing and hence choose to employ walking aids. In contrast, the study revealed that individuals who exhibited toe walking tendencies were often of a younger age compared to those who did not engage in such walking behavior (25). Clinicians and therapists may find it useful to better comprehend the relationship between a patient’s movement patterns and their capacity for balance and gait in order to determine the optimal course of treatment for each individual patient with HSP. The treatments employed may include ankle-foot surgery in cases with structural ankle-foot abnormalities, the use of balance-assistive devices, and the incorporation of Virtual Reality (VR) games. These interventions aim to decrease dependence on hip strategies. The application of virtual reality (VR) can be employed as a therapeutic approach for the treatment of these individuals, as it serves to reinforce the connections between the vestibular and visual systems, while also improving both static and dynamic postural stability. Virtual reality (VR) has the potential to aid patients in restoring self-confidence, promoting increased autonomy in doing daily tasks, mitigating anxiety, and improving social relations through the improvement of balance (31). The existing literature suggests that this therapeutic approach offers various advantages, such as enhancing locomotion patterns, balance and posture, upper and lower limb functions, as well as fostering patient motivation for engaging in exercise (32). Bruin et al. assert that physical exercise regimens incorporating virtual reality games offer a multitude of benefits in comparison to traditional balance training methods (33). For example, different scenarios and therapeutic protocols can be customized to accommodate the individual patient’s specific needs and interests. This customization facilitates enhancements in both balance and motor coordination, while also fostering motor learning by inducing alterations in cerebral architecture. Consequently, patients’ reliance on and inclination towards engaging in physical exercise are heightened. There is a lack of published randomized or nonrandomized research investigating the use of virtual reality (VR) in individuals with HSP within the PubMed database until the year 2023. Zeigelboim et al. recruited a sample of forty patients diagnosed with unspecified form of HSP for a randomized controlled clinical intervention that utilized the Wii^®^ system, Wii-Remote, and Wii Balance Board developed by Nintendo. In both cohorts, a duration of 30 min of virtual reality (VR) games was allocated twice each week, resulting in a cumulative total of 20 sessions. In the present study, Group I engaged in a total of five balance games. Group II engaged in a series of five balancing games, followed by an extra four strength games. The selection of these games was intended to facilitate the promotion of postural instability and alterations in balance. Upon completion of data collection, Group I will be provided with access to strength training activities in order to guarantee that both groups get comparable rehabilitative interventions (10). The training phases were conducted concurrently to avoid delays and modifications that would compromise the effectiveness of the instruction. Following the 20 intervention sessions, a second assessment was undertaken.

## 4 Discussion

Currently, there is a significant emphasis on the implementation of technologies for gene editing in order to develop customised medical treatments for individuals with distinct genetic patterns, however achievement of the desired outcome in HSP is really challenging as it necessitates the precise targeting of 80 genes. It is worth considering that certain individuals may exhibit one of several causative mutations or two confounding heterozygous recessive conditions (34). Hence, it is crucial to explore non-pharmacological interventions, such as physiotherapy, in order to enhance the potential for improved outcomes. Although certain drugs, such as progabide, botulinum toxin injection, gabapentin, and l-dopa, demonstrate promising outcomes (35, 36), there currently remains a lack of comprehensive data regarding the effectiveness of physiotherapy. Consequently, we decided to conduct a critical review of the available evidence focusing exclusively to the therapeutic advantages associated with various forms of physical therapy in the context of HSP, emphasizing the specific benefit of every distinct approach in relation to muscle relaxation, muscle strength, spasticity reduction, improvement of weakness, enhancement of balance, posture, walking ability, and overall quality of life. The available evidence regarding the effectiveness of physical therapies in individuals with HSP is currently quite limited. This evidence primarily consists of uncontrolled studies and case reports. To date, there exists a single case report that suggests the potential effectiveness of electrical stimulation in enhancing motor neuron functionality, specifically in a 26 years-old male individual. The implementation of trilateral stimulation of the quadriceps and anterior musculature, conducted on a bilateral basis, over a period of 3 months, has been found to enhance the outcome of gait analysis performance. It is important to note that the evaluation of this particular case report has resulted in a significantly low rating (37). A range of robotic locomotive systems are presently employed to enhance the gait of individuals suffering from neurological disorders. A single uncontrolled study has demonstrated the advantageous impact of utilizing robotics assistance in intensive training for adults affected by pure HSP. The scores of the Berg balance and meters walk tests exhibited significant improvement as a result of the implementation of robotic aided training (13). Currently, there is a lack of substantial evidence to support the efficacy of particular physical training exercises and their optimal timing for individuals with HSP. Nevertheless, a single study has documented the advantages of engaging in a comprehensive regimen consisting of strength training, stretching exercises, and fundamental functional exercises. This intervention was conducted over a period of 8 weeks, with a daily time commitment of 90 min (24). A small, uncontrolled trial was conducted to evaluate the effectiveness of hydro-training in improving locomotor function among individuals with late-onset HSP following a 10 weeks intervention consisting of 45 min sessions. A notable enhancement was observed in the kinematics and spatiotemporal measures aimed at enhancing gait. Nevertheless, additional research is necessary to establish the effectiveness of hydro-training (14). Currently, the absence of sufficient data regarding the enhancement of precision parameters prevents the provision of definitive statements. It is possible to inferred that the integration of physiotherapy and medical therapy offers the most optimal resolution currently available (34). However, it is imperative to conduct studies on larger cohorts in order to provide conclusive findings for management and therapeutic strategies.

## 5 Conclusion

**Figure 2:**
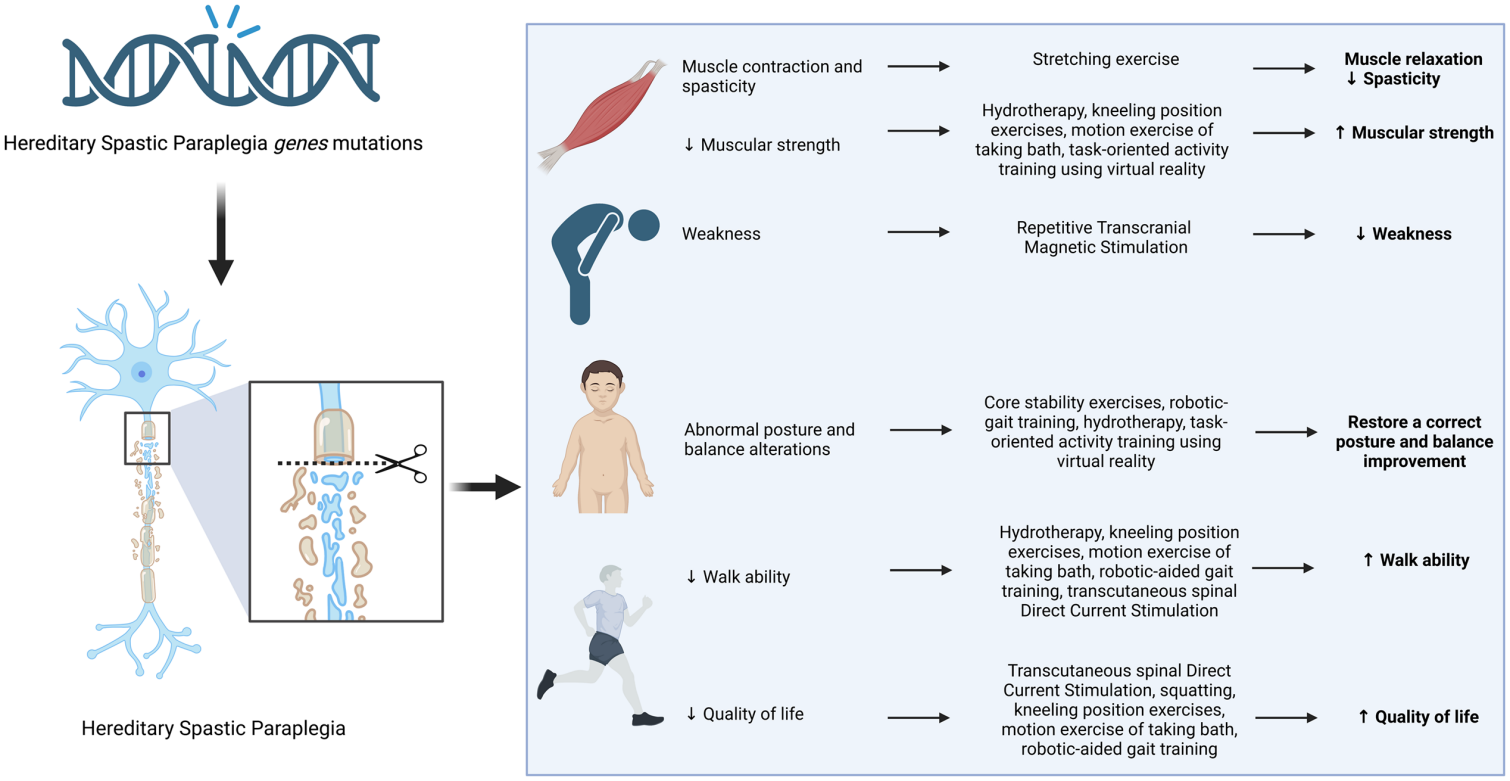
Physiotherapy interventions in Hereditary Spastic Paraplegia.

Recent advances in next-generation sequencing technologies have revealed the complexity of neurodevelopmental disorders (NDDs), revealing the involvement of genes related to synaptic plasticity, chromatin remodelers, transcription regulation, and cytoskeleton organization (38–42). This has led to advancements in clinical phenotype refinement, predictive information, and targeted therapeutics (43–45). On the other hand, there seems to be a lack of progress in generating innovative physical therapy suggestions. There exists potential for the implementation of several therapeutic interventions, including electrostimulation, magnetotherapy, hydrotherapy, physical therapy, robot-assisted gait training, and balance rehabilitation, to enhance muscle strength, alleviate spasticity, increase balancing capacity, enhance walking ability, and ultimately enhance the overall quality of life for those diagnosed with HSP (Figure 2 and Table 2). Nevertheless, additional investigation with more extensive sample sizes is required in order to validate their efficacy.

**Table 2:** Recommended interventions in patients with hereditary spastic paraplegia.

Purpose	Therapeutic approach	Levels of evidence	GRADE of recommendation
Muscle relaxation	Stretching exercises	II II	B B
Muscle strength improvement	Hydrotherapy; kneeling position exercises; motion exercise of taking bath; task-oriented activity training using virtual reality	V	B
Spasticity reduction	Repetitive Transcranial Magnetic Stimulation; stretching exercises; transcutaneous spinal Direct Current Stimulation	II II	B B
Weakness improvement	Repetitive Transcranial Magnetic Stimulation	II	B
Balance capacity and posture improvement	Core stability exercises; robotic-aided gait training; hydrotherapy; task-oriented activity training using virtual reality	II II II	B B B
Walk ability improvement	Hydrotherapy; squatting; kneeling position exercises; motion exercise of taking bath; robotic-aided gait training; transcutaneous spinal direct current stimulation	II II II	B B B
Quality of life improvement	Transcutaneous spinal direct current stimulation; squatting; kneeling position exercises; motion exercise of taking bath; robotic-aided gait training	II II V	B B B

Physiotherapy (PT) for muscular relaxation, spasticity reduction, weakness improvement, balance and posture improvement, and walk ability improvement has received B recommendations in level II research. Instead, PT has been recommended with grades of B in studies with levels of evidence II and V for quality of life and B for muscle strength in a single study with level of evidence V.

## Author contributions

ADL: Writing - original draft and realized images and tables. FCi: Writing - original draft. SLB: Writing - original draft. GS: Writing - original draft. FCh: Writing - reviewed & editing. GF: Writing - original draft.
